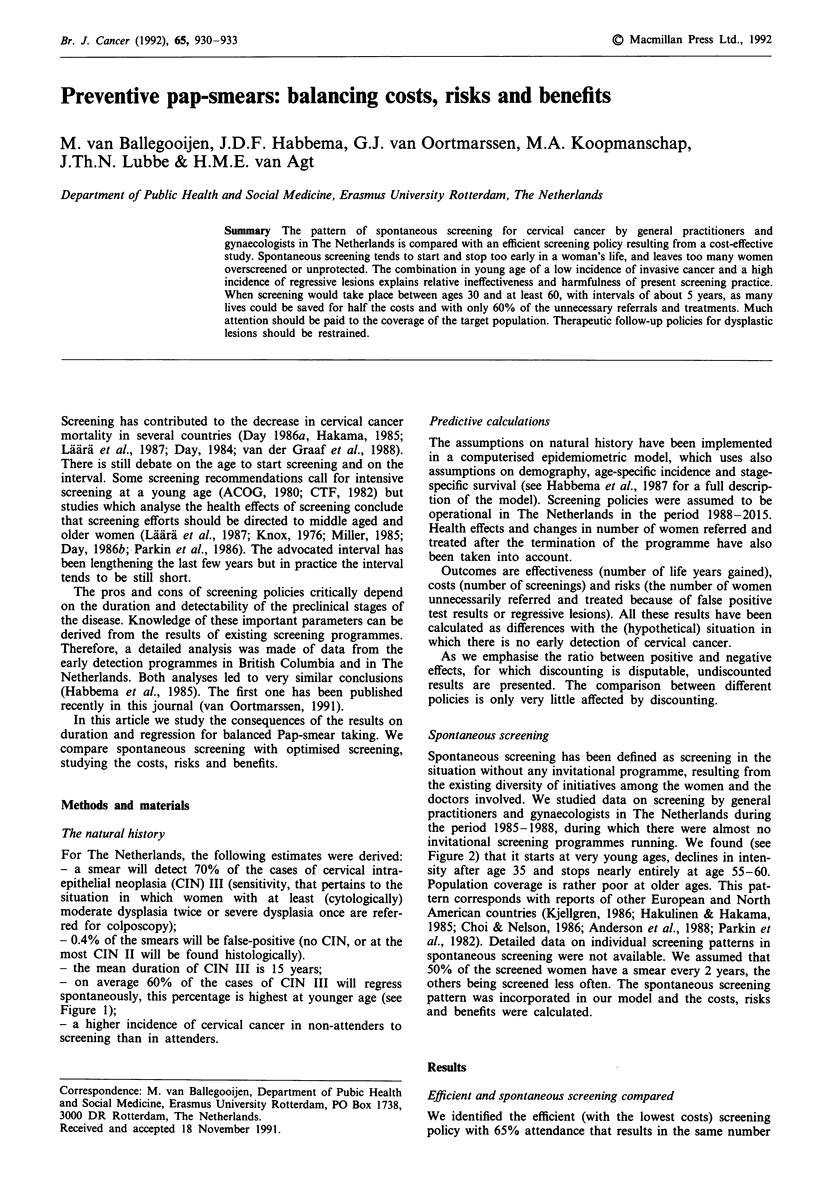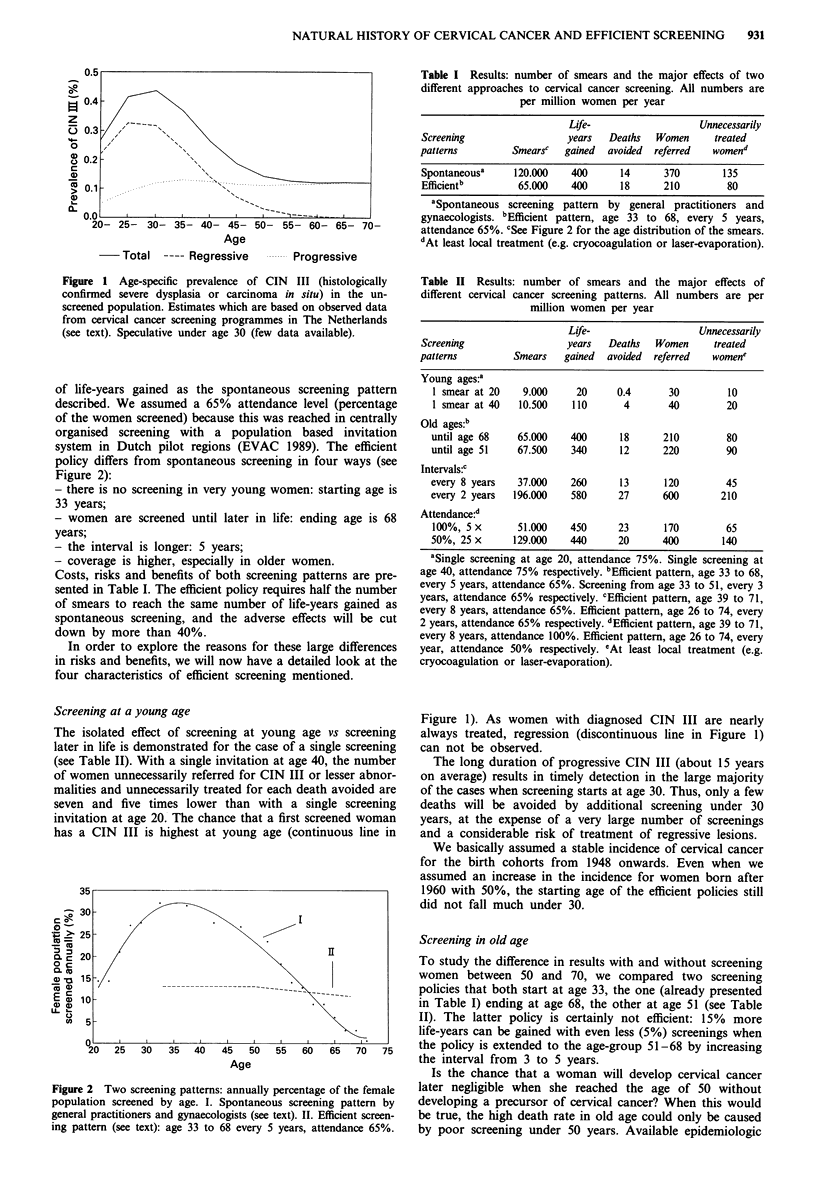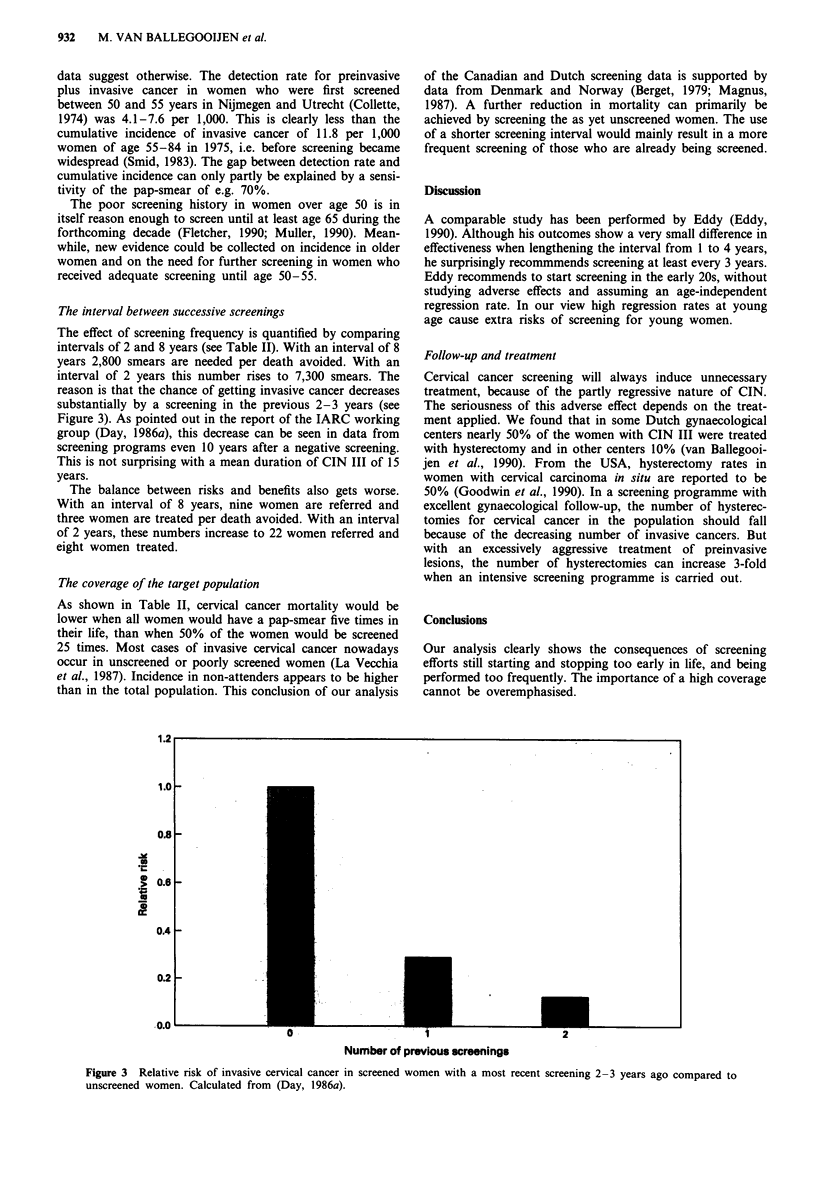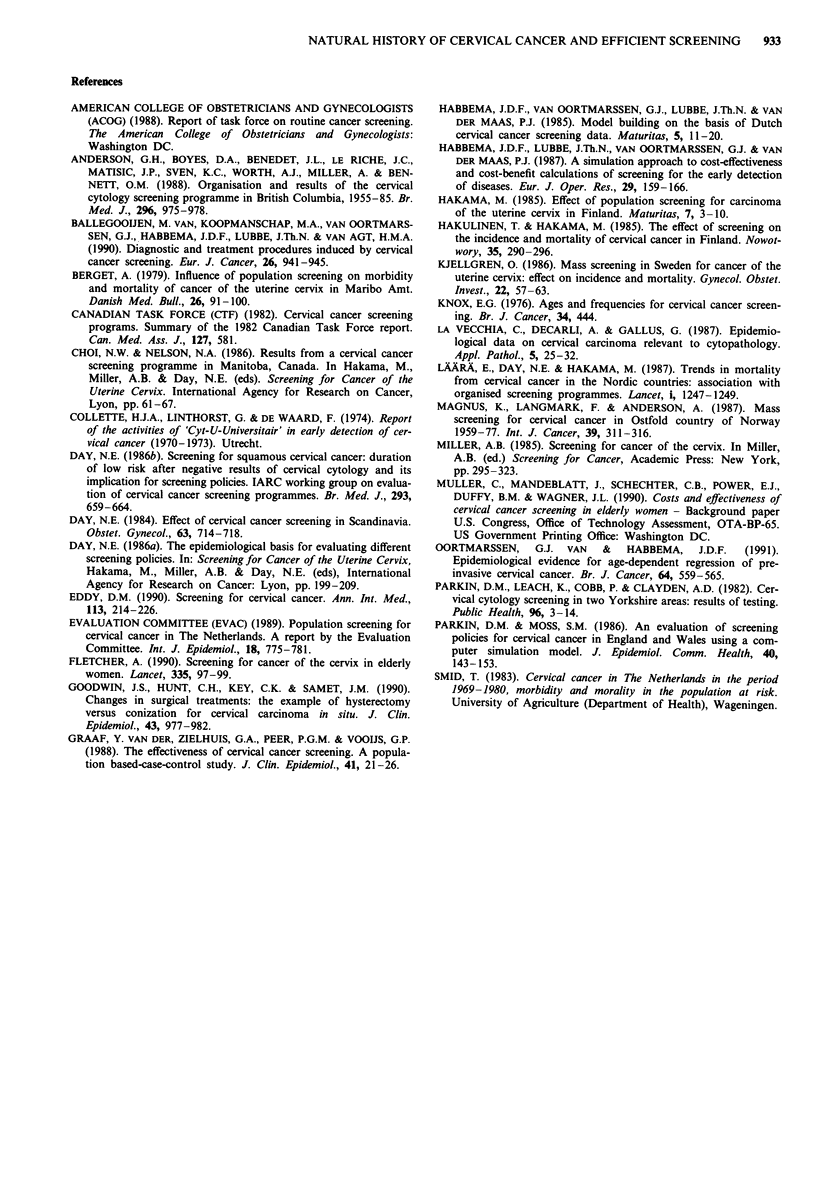# Preventive Pap-smears: balancing costs, risks and benefits.

**DOI:** 10.1038/bjc.1992.195

**Published:** 1992-06

**Authors:** M. van Ballegooijen, J. D. Habbema, G. J. van Oortmarssen, M. A. Koopmanschap, J. T. Lubbe, H. M. van Agt

**Affiliations:** Department of Public Health and Social Medicine, Erasmus University Rotterdam, The Netherlands.

## Abstract

The pattern of spontaneous screening for cervical cancer by general practitioners and gynaecologists in The Netherlands is compared with an efficient screening policy resulting from a cost-effective study. Spontaneous screening tends to start and stop too early in a woman's life, and leaves too many women overscreened or unprotected. The combination in young age of a low incidence of invasive cancer and a high incidence of regressive lesions explains relative ineffectiveness and harmfulness of present screening practice. When screening would take place between ages 30 and at least 60, with intervals of about 5 years, as many lives could be saved for half the costs and with only 60% of the unnecessary referrals and treatments. Much attention should be paid to the coverage of the target population. Therapeutic follow-up policies for dysplastic lesions should be restrained.


*Br. J. Cancer* (1992), **65**, 930–933 © Macmillan Press Ltd., 1992

### Preventive pap-smears: balancing costs, risks and benefits

M. van Ballegooijen, J.D.F. Habbema, G.J. van Oortmarssen, M.A. Koopmanschap, J.Th.N. Lubbe & H.M.E. van Agt

*Department of Public Health and Social Medicine, Erasmus University Rotterdam, The Netherlands*

**Summary** The pattern of spontaneous screening for cervical cancer by general practitioners and gynaecologists in The Netherlands is compared with an efficient screening policy resulting from a cost-effective study. Spontaneous screening tends to start and stop too early in a woman's life, and leaves too many women overscreened or unprotected. The combination in young age of a low incidence of invasive cancer and a high incidence of regressive lesions explains relative ineffectiveness and harmfulness of present screening practice. When screening would take place between ages 30 and at least 60, with intervals of about 5 years, as many lives could be saved for half the costs and with only 60% of the unnecessary referrals and treatments. Much attention should be paid to the coverage of the target population. Therapeutic follow-up policies for dysplastic lesions should be restrained.

Screening has contributed to the decrease in cervical cancer mortality in several countries (Day 1986*a*, Hakama, 1985; Läärä *et al*., 1987; Day, 1984; van der Graaf *et al*., 1988). There is still debate on the age to start screening and on the interval. Some screening recommendations call for intensive screening at a young age (ACOG, 1980; CTF, 1982) but studies which analyse the health effects of screening conclude that screening efforts should be directed to middle aged and older women (Läärä *et al*., 1987; Knox, 1976; Miller, 1985; Day, 1986*b*; Parkin *et al*., 1986). The advocated interval has been lengthening the last few years but in practice the interval tends to be still short.

The pros and cons of screening policies critically depend on the duration and detectability of the preclinical stages of the disease. Knowledge of these important parameters can be derived from the results of existing screening programmes. Therefore, a detailed analysis was made of data from the early detection programmes in British Columbia and in The Netherlands. Both analyses led to very similar conclusions (Habbema *et al*., 1985). The first one has been published recently in this journal (van Oortmarssen, 1991).

In this article we study the consequences of the results on duration and regression for balanced Pap-smear taking. We compare spontaneous screening with optimised screening, studying the costs, risks and benefits.

### Methods and materials

### *The natural history*

For The Netherlands, the following estimates were derived:
- a smear will detect 70% of the cases of cervical intra-epithelial neoplasia (CIN) III (sensitivity, that pertains to the situation in which women with at least (cytologically) moderate dysplasia twice or severe dysplasia once are referred for colposcopy);
- 0.4% of the smears will be false-positive (no CIN, or at the most CIN II will be found histologically).
- the mean duration of CIN III is 15 years;
- on average 60% of the cases of CIN III will regress spontaneously, this percentage is highest at younger age (see Figure 1);
- a higher incidence of cervical cancer in non-attenders to screening than in attenders.

Correspondence: M. van Ballegooijen, Department of Pubic Health and Social Medicine, Erasmus University Rotterdam, PO Box 1738, 3000 DR Rotterdam, The Netherlands.
Received and accepted 18 November 1991.

### *Predictive calculations*

The assumptions on natural history have been implemented in a computerised epidemiometric model, which uses also assumptions on demography, age-specific incidence and stage-specific survival (see Habbema *et al*., 1987 for a full description of the model). Screening policies were assumed to be operational in The Netherlands in the period 1988–2015. Health effects and changes in number of women referred and treated after the termination of the programme have also been taken into account.

Outcomes are effectiveness (number of life years gained), costs (number of screenings) and risks (the number of women unnecessarily referred and treated because of false positive test results or regressive lesions). All these results have been calculated as differences with the (hypothetical) situation in which there is no early detection of cervical cancer.

As we emphasise the ratio between positive and negative effects, for which discounting is disputable, undiscounted results are presented. The comparison between different policies is only very little affected by discounting.

### *Spontaneous screening*

Spontaneous screening has been defined as screening in the situation without any invitational programme, resulting from the existing diversity of initiatives among the women and the doctors involved. We studied data on screening by general practitioners and gynaecologists in The Netherlands during the period 1985–1988, during which there were almost no invitational screening programmes running. We found (see Figure 2) that it starts at very young ages, declines in intensity after age 35 and stops nearly entirely at age 55–60. Population coverage is rather poor at older ages. This pattern corresponds with reports of other European and North American countries (Kjellgren, 1986; Hakulinen & Hakama, 1985; Choi & Nelson, 1986; Anderson *et al*., 1988; Parkin *et al*., 1982). Detailed data on individual screening patterns in spontaneous screening were not available. We assumed that 50% of the screened women have a smear every 2 years, the others being screened less often. The spontaneous screening pattern was incorporated in our model and the costs, risks and benefits were calculated.

### Results

### *Efficient and spontaneous screening compared*

We identified the efficient (with the lowest costs) screening policy with 65% attendance that results in the same number

**Figure 1** Age-specific prevalence of CIN III (histologically confirmed severe dysplasia or carcinoma *in situ*) in the unscreened population. Estimates which are based on observed data from cervical cancer screening programmes in The Netherlands (see text). Speculative under age 30 (few data available).

of life-years gained as the spontaneous screening pattern described. We assumed a 65% attendance level (percentage of the women screened) because this was reached in centrally organised screening with a population based invitation system in Dutch pilot regions (EVAC 1989). The efficient policy differs from spontaneous screening in four ways (see Figure 2):

- there is no screening in very young women: starting age is 33 years;
- women are screened until later in life: ending age is 68 years;
- the interval is longer: 5 years;
- coverage is higher, especially in older women.

Costs, risks and benefits of both screening patterns are presented in Table I. The efficient policy requires half the number of smears to reach the same number of life-years gained as spontaneous screening, and the adverse effects will be cut down by more than 40%.

In order to explore the reasons for these large differences in risks and benefits, we will now have a detailed look at the four characteristics of efficient screening mentioned.

*Screening at a young age*

The isolated effect of screening at young age *vs* screening later in life is demonstrated for the case of a single screening (see Table II). With a single invitation at age 40, the number of women unnecessarily referred for CIN III or lesser abnormalities and unnecessarily treated for each death avoided are seven and five times lower than with a single screening invitation at age 20. The chance that a first screened woman has a CIN III is highest at young age (continuous line in

**Figure 2** Two screening patterns: annually percentage of the female population screened by age. I. Spontaneous screening pattern by general practitioners and gynaecologists (see text). II. Efficient screening pattern (see text): age 33 to 68 every 5 years, attendance 65%.

**Table I** Results: number of smears and the major effects of two different approaches to cervical cancer screening. All numbers are per million women per year

| *Screening patterns* | *Smears*[c] | *Life-years gained* | *Deaths avoided* | *Women referred* | *Unnecessarily treated women*[d] |
|---|---|---|---|---|---|
| Spontaneous[a] | 120.000 | 400 | 14 | 370 | 135 |
| Efficient[b] | 65.000 | 400 | 18 | 210 | 80 |

[a]Spontaneous screening pattern by general practitioners and gynaecologists. [b]Efficient pattern, age 33 to 68, every 5 years, attendance 65%. [c]See Figure 2 for the age distribution of the smears. [d]At least local treatment (e.g. cryocoagulation or laser-evaporation).

**Table II** Results: number of smears and the major effects of different cervical cancer screening patterns. All numbers are per million women per year

| *Screening patterns* | *Smears* | *Life-years gained* | *Deaths avoided* | *Women referred* | *Unnecessarily treated women*[e] |
|---|---|---|---|---|---|
| Young ages:[a] | | | | | |
| 1 smear at 20 | 9.000 | 20 | 0.4 | 30 | 10 |
| 1 smear at 40 | 10.500 | 110 | 4 | 40 | 20 |
| Old ages:[b] | | | | | |
| until age 68 | 65.000 | 400 | 18 | 210 | 80 |
| until age 51 | 67.500 | 340 | 12 | 220 | 90 |
| Intervals:[c] | | | | | |
| every 8 years | 37.000 | 260 | 13 | 120 | 45 |
| every 2 years | 196.000 | 580 | 27 | 600 | 210 |
| Attendance:[d] | | | | | |
| 100%, 5 × | 51.000 | 450 | 23 | 170 | 65 |
| 50%, 25 × | 129.000 | 440 | 20 | 400 | 140 |

[a]Single screening at age 20, attendance 75%. Single screening at age 40, attendance 75% respectively. [b]Efficient pattern, age 33 to 68, every 5 years, attendance 65%. Screening from age 33 to 51, every 3 years, attendance 65% respectively. [c]Efficient pattern, age 39 to 71, every 8 years, attendance 65%. Efficient pattern, age 26 to 74, every 2 years, attendance 65% respectively. [d]Efficient pattern, age 39 to 71, every 8 years, attendance 100%. Efficient pattern, age 26 to 74, every year, attendance 50% respectively. [e]At least local treatment (e.g. cryocoagulation or laser-evaporation).

Figure 1). As women with diagnosed CIN III are nearly always treated, regression (discontinuous line in Figure 1) can not be observed.

The long duration of progressive CIN III (about 15 years on average) results in timely detection in the large majority of the cases when screening starts at age 30. Thus, only a few deaths will be avoided by additional screening under 30 years, at the expense of a very large number of screenings and a considerable risk of treatment of regressive lesions.

We basically assumed a stable incidence of cervical cancer for the birth cohorts from 1948 onwards. Even when we assumed an increase in the incidence for women born after 1960 with 50%, the starting age of the efficient policies still did not fall much under 30.

*Screening in old age*

To study the difference in results with and without screening women between 50 and 70, we compared two screening policies that both start at age 33, the one (already presented in Table I) ending at age 68, the other at age 51 (see Table II). The latter policy is certainly not efficient: 15% more life-years can be gained with even less (5%) screenings when the policy is extended to the age-group 51–68 by increasing the interval from 3 to 5 years.

Is the chance that a woman will develop cervical cancer later negligible when she reached the age of 50 without developing a precursor of cervical cancer? When this would be true, the high death rate in old age could only be caused by poor screening under 50 years. Available epidemiologic data suggest otherwise. The detection rate for preinvasive plus invasive cancer in women who were first screened between 50 and 55 years in Nijmegen and Utrecht (Collette, 1974) was 4.1–7.6 per 1,000. This is clearly less than the cumulative incidence of invasive cancer of 11.8 per 1,000 women of age 55–84 in 1975, i.e. before screening became widespread (Smid, 1983). The gap between detection rate and cumulative incidence can only partly be explained by a sensitivity of the pap-smear of e.g. 70%.

The poor screening history in women over age 50 is in itself reason enough to screen until at least age 65 during the forthcoming decade (Fletcher, 1990; Muller, 1990). Meanwhile, new evidence could be collected on incidence in older women and on the need for further screening in women who received adequate screening until age 50–55.

### *The interval between successive screenings*

The effect of screening frequency is quantified by comparing intervals of 2 and 8 years (see Table II). With an interval of 8 years 2,800 smears are needed per death avoided. With an interval of 2 years this number rises to 7,300 smears. The reason is that the chance of getting invasive cancer decreases substantially by a screening in the previous 2–3 years (see Figure 3). As pointed out in the report of the IARC working group (Day, 1986*a*), this decrease can be seen in data from screening programs even 10 years after a negative screening. This is not surprising with a mean duration of CIN III of 15 years.

The balance between risks and benefits also gets worse. With an interval of 8 years, nine women are referred and three women are treated per death avoided. With an interval of 2 years, these numbers increase to 22 women referred and eight women treated.

### *The coverage of the target population*

As shown in Table II, cervical cancer mortality would be lower when all women would have a pap-smear five times in their life, than when 50% of the women would be screened 25 times. Most cases of invasive cervical cancer nowadays occur in unscreened or poorly screened women (La Vecchia *et al.*, 1987). Incidence in non-attenders appears to be higher than in the total population. This conclusion of our analysis of the Canadian and Dutch screening data is supported by data from Denmark and Norway (Berget, 1979; Magnus, 1987). A further reduction in mortality can primarily be achieved by screening the as yet unscreened women. The use of a shorter screening interval would mainly result in a more frequent screening of those who are already being screened.

### Discussion

A comparable study has been performed by Eddy (Eddy, 1990). Although his outcomes show a very small difference in effectiveness when lengthening the interval from 1 to 4 years, he surprisingly recommmends screening at least every 3 years. Eddy recommends to start screening in the early 20s, without studying adverse effects and assuming an age-independent regression rate. In our view high regression rates at young age cause extra risks of screening for young women.

### *Follow-up and treatment*

Cervical cancer screening will always induce unnecessary treatment, because of the partly regressive nature of CIN. The seriousness of this adverse effect depends on the treatment applied. We found that in some Dutch gynaecological centers nearly 50% of the women with CIN III were treated with hysterectomy and in other centers 10% (van Ballegooijen *et al.*, 1990). From the USA, hysterectomy rates in women with cervical carcinoma *in situ* are reported to be 50% (Goodwin *et al.*, 1990). In a screening programme with excellent gynaecological follow-up, the number of hysterectomies for cervical cancer in the population should fall because of the decreasing number of invasive cancers. But with an excessively aggressive treatment of preinvasive lesions, the number of hysterectomies can increase 3-fold when an intensive screening programme is carried out.

### Conclusions

Our analysis clearly shows the consequences of screening efforts still starting and stopping too early in life, and being performed too frequently. The importance of a high coverage cannot be overemphasised.

**Figure 3** Relative risk of invasive cervical cancer in screened women with a most recent screening 2–3 years ago compared to unscreened women. Calculated from (Day, 1986*a*).

### References

AMERICAN COLLEGE OF OBSTETRICIANS AND GYNECOLOGISTS (ACOG) (1988). Report of task force on routine cancer screening. *The American College of Obstetricians and Gynecologists*: Washington DC.

ANDERSON, G.H., BOYES, D.A., BENEDET, J.L., LE RICHE, J.C., MATISIC, J.P., SVEN, K.C., WORTH, A.J., MILLER, A. & BENNETT, O.M. (1988). Organisation and results of the cervical cytology screening programme in British Columbia, 1955–85. *Br. Med. J.*, **296**, 975–978.

BALLEGOOIJEN, M. VAN, KOOPMANSCHAP, M.A., VAN OORTMARSSEN, G.J., HABBEMA, J.D.F., LUBBE, J.Th.N. & VAN AGT, H.M.A. (1990). Diagnostic and treatment procedures induced by cervical cancer screening. *Eur. J. Cancer*, **26**, 941–945.

BERGET, A. (1979). Influence of population screening on morbidity and mortality of cancer of the uterine cervix in Maribo Amt. *Danish Med. Bull.*, **26**, 91–100.

CANADIAN TASK FORCE (CTF) (1982). Cervical cancer screening programs. Summary of the 1982 Canadian Task Force report. *Can. Med. Ass. J.*, **127**, 581.

CHOI, N.W. & NELSON, N.A. (1986). Results from a cervical cancer screening programme in Manitoba, Canada. In Hakama, M., Miller, A.B. & Day, N.E. (eds). *Screening for Cancer of the Uterine Cervix*. International Agency for Research on Cancer, Lyon, pp. 61–67.

COLLETTE, H.J.A., LINTHORST, G. & DE WAARD, F. (1974). *Report of the activities of 'Cyt-U-Universitair' in early detection of cervical cancer* (1970–1973). Utrecht.

DAY, N.E. (1986*b*). Screening for squamous cervical cancer: duration of low risk after negative results of cervical cytology and its implication for screening policies. IARC working group on evaluation of cervical cancer screening programmes. *Br. Med. J.*, **293**, 659–664.

DAY, N.E. (1984). Effect of cervical cancer screening in Scandinavia. *Obstet. Gynecol.*, **63**, 714–718.

DAY, N.E. (1986*a*). The epidemiological basis for evaluating different screening policies. In: *Screening for Cancer of the Uterine Cervix*, Hakama, M., Miller, A.B. & Day, N.E. (eds), International Agency for Research on Cancer: Lyon, pp. 199–209.

EDDY, D.M. (1990). Screening for cervical cancer. *Ann. Int. Med.*, **113**, 214–226.

EVALUATION COMMITTEE (EVAC) (1989). Population screening for cervical cancer in The Netherlands. A report by the Evaluation Committee. *Int. J. Epidemiol.*, **18**, 775–781.

FLETCHER, A. (1990). Screening for cancer of the cervix in elderly women. *Lancet*, **335**, 97–99.

GOODWIN, J.S., HUNT, C.H., KEY, C.K. & SAMET, J.M. (1990). Changes in surgical treatments: the example of hysterectomy versus conization for cervical carcinoma *in situ*. *J. Clin. Epidemiol.*, **43**, 977–982.

GRAAF, Y. VAN DER, ZIELHUIS, G.A., PEER, P.G.M. & VOOIJS, G.P. (1988). The effectiveness of cervical cancer screening. A population based-case-control study. *J. Clin. Epidemiol.*, **41**, 21–26.

HABBEMA, J.D.F., VAN OORTMARSSEN, G.J., LUBBE, J.Th.N. & VAN DER MAAS, P.J. (1985). Model building on the basis of Dutch cervical cancer screening data. *Maturitas*, **5**, 11–20.

HABBEMA, J.D.F., LUBBE, J.Th.N., VAN OORTMARSSEN, G.J. & VAN DER MAAS, P.J. (1987). A simulation approach to cost-effectiveness and cost-benefit calculations of screening for the early detection of diseases. *Eur. J. Oper. Res.*, **29**, 159–166.

HAKAMA, M. (1985). Effect of population screening for carcinoma of the uterine cervix in Finland. *Maturitas*, **7**, 3–10.

HAKULINEN, T. & HAKAMA, M. (1985). The effect of screening on the incidence and mortality of cervical cancer in Finland. *Nowotwory*, **35**, 290–296.

KJELLGREN, O. (1986). Mass screening in Sweden for cancer of the uterine cervix: effect on incidence and mortality. *Gynecol. Obstet. Invest.*, **22**, 57–63.

KNOX, E.G. (1976). Ages and frequencies for cervical cancer screening. *Br. J. Cancer*, **34**, 444.

LA VECCHIA, C., DECARLI, A. & GALLUS, G. (1987). Epidemiological data on cervical carcinoma relevant to cytopathology. *Appl. Pathol.*, **5**, 25–32.

LÄÄRÄ, E., DAY, N.E. & HAKAMA, M. (1987). Trends in mortality from cervical cancer in the Nordic countries: association with organised screening programmes. *Lancet*, **i**, 1247–1249.

MAGNUS, K., LANGMARK, F. & ANDERSON, A. (1987). Mass screening for cervical cancer in Ostfold country of Norway 1959–77. *Int. J. Cancer*, **39**, 311–316.

MILLER, A.B. (1985). Screening for cancer of the cervix. In Miller, A.B. (ed.) *Screening for Cancer*, Academic Press: New York, pp. 295–323.

MULLER, C., MANDEBLATT, J., SCHECHTER, C.B., POWER, E.J., DUFFY, B.M. & WAGNER, J.L. (1990). *Costs and effectiveness of cervical cancer screening in elderly women* – Background paper U.S. Congress, Office of Technology Assessment, OTA-BP-65. US Government Printing Office: Washington DC.

OORTMARSSEN, G.J. VAN & HABBEMA, J.D.F. (1991). Epidemiological evidence for age-dependent regression of pre-invasive cervical cancer. *Br. J. Cancer*, **64**, 559–565.

PARKIN, D.M., LEACH, K., COBB, P. & CLAYDEN, A.D. (1982). Cervical cytology screening in two Yorkshire areas: results of testing. *Public Health*, **96**, 3–14.

PARKIN, D.M. & MOSS, S.M. (1986). An evaluation of screening policies for cervical cancer in England and Wales using a computer simulation model. *J. Epidemiol. Comm. Health*, **40**, 143–153.

SMID, T. (1983). *Cervical cancer in The Netherlands in the period 1969–1980, morbidity and morality in the population at risk*. University of Agriculture (Department of Health), Wageningen.